# Multi-parametric MRI without artificial erection for preoperative assessment of primary penile carcinoma: A pilot study on the correlation between imaging and histopathological findings

**DOI:** 10.1016/j.ejro.2023.100478

**Published:** 2023-01-28

**Authors:** Marta D. Switlyk, Andreas Hopland, Shivanthe Sivanesan, Bjørn Brennhovd, Fredrik Ottosson, Kjetil Berner, Ulrika Axcrona, Knut H. Hole

**Affiliations:** a Department of Radiology, The Norwegian Radium Hospital, Oslo University Hospital, Oslo, Norway; b Department of Urology, The Norwegian Radium Hospital, Oslo University Hospital, Oslo, Norway; c Institute of Clinical Medicine (KlinMED), Faculty of Medicine, University of Oslo, Oslo, Norway; d Department of Genitourinary Oncology, The Norwegian Radium Hospital, Oslo University Hospital, Oslo, Norway; e Department of Pathology, The Norwegian Radium Hospital, Oslo University Hospital, Oslo, Norway

**Keywords:** Primary penile carcinoma, Surgery, Multi-parametric MRI, Histopathology

## Abstract

**Purpose:**

We aimed to evaluate the diagnostic potential of non-erectile multi-parametric magnetic resonance imaging (mpMRI) for preoperative assessment of primary penile squamous cell carcinoma (SCC).

**Method:**

Twenty-five patients who underwent surgery for penile SCC were included. Preoperative mpMRI without artificial erection was performed in all patients. The preoperative MRI protocol consisted of high-resolution morphological and functional sequences (diffusion-weighted imaging and dynamic contrast-enhanced MRI perfusion) covering the penis and lower pelvis. T and N staging, according to the 8th edition of the Union for International Cancer Control TNM classification, as well as the largest diameter and thickness/infiltration depth of the primary lesions were determined in all patients. Imaging data were retrospectively collected and compared with the final histopathology reports.

**Results:**

Very good agreement was observed between MRI and histopathology for the involvement of corpus spongiosum (*p* = 0.002) and good agreement was observed for the involvement of penile urethra and tunica albuginea/corpus cavernosum (*p* < 0.001 and *p* = 0.007, respectively). Good agreement was observed between MRI and histopathology for overall T staging and weaker, but still good agreement was observed for N staging (*p* < 0.001 and *p* = 0.002, respectively). A strong and significant correlation was observed between MRI and histopathology for the largest diameter and thickness/infiltration depth of the primary lesions (*p* < 0.001).

**Conclusions:**

Good concordance was observed between MRI and histopathological findings. Our initial findings indicate that non-erectile mpMRI is useful in preoperative assessment of primary penile SCC.

## Highlights

• An appropriate preoperative evaluation of penile cancer is important to optimize the clinical management.
• Good concordance was observed between imaging and histopathological findings.
• Non-erectile mpMRI allows precise assessment of penile tumors.

## 1 Introduction

Primary malignant epithelial tumors of the penis are rare. A wide variation is observed in the prevalence of penile cancer, ranging from up to 6.8 per 100,000 in Africa, Asia, and South America to less than 1 per 100,000 in North America and Europe [1], [2]. Squamous cell carcinoma (SCC) is the most common penile malignancy [3]. Penile amputative surgery is associated with significant functional, sexual and psychological deficits, despite high oncological control rates [4], [5]. Recently, organ-sparing techniques have become more common as they offer improvements in functional outcomes, quality of life, body image, and well-being [4], [5], [6], [7]. However, there is a potential for increased risk of local recurrence after organ-sparing surgery (OSS) compared with amputation of the penis [4], [8]. Thorough preoperative staging can improve patient selection and decrease the recurrence rate.

Current clinical guidelines for the diagnosis and staging of penile cancer recommend the use of magnetic resonance imaging (MRI) in cases intended for OSS [9]. However, this recommendation is rated as weak, and the results of most of the studies supporting this recommendation are based on morphological MRI alone [2], [10], [11]. Thus, the potential of functional MRI sequences has not yet been investigated. Moreover, artificial erection is suggested for performing MRI, although this invasive technique is not used routinely in clinical practice and there are some important contraindications and side effects of this method [12], [13]. Pain associated with this procedure may be an important limitation in patients with invasive penile carcinoma and several patients may experience the embarrassment and discomfort during the procedure.

In recent years, MRI has evolved to become the gold standard in oncological imaging, providing excellent anatomical information as well as molecular and physiological information in vivo using diffusion-weighted imaging (DWI) and dynamic contrast-enhanced (DCE) MRI perfusion [14]. Multi-parametric MRI (mpMRI) allows integrated evaluation of morphological images and at least two functional sequences (DWI and DCE-MRI), and has been successfully preformed in several malignancies such as prostate cancer [15]. DCE-MRI provides information regarding tumor vascularity and hemodynamics, while DWI allows non-invasive characterization of biological tissues based on the measurements of random microscopic motion of water protons (Brownian motion) [16], [17]. The ability to detect tumors on DWI is based on the premise that tumors have increased cellularity compared to the background tissue [16]. Consequently, the contrast is accentuated by high signal intensity in the cellular tumor and low signal intensity in the normal background tissue [16]. Although data are lacking, the use of DCE-MRI perfusion and DWI in the staging of penile cancers can potentially aid in a more precise determination of the infiltration depth, identification of small lesions, and further tissue characterization [12]. MpMRI can also be useful in accurately outlining the proximal tumor extent and tumor satellites, thereby assessing the feasibility of organ-sparing surgery or partial penectomy [12].

In summary, MRI is a promising diagnostic tool for the preoperative staging of penile cancer. This study aimed to assess the concordance between imaging and histopathological features and to evaluate the diagnostic potential of mpMRI without artificial erection in preoperative assessment of primary penile carcinoma.

## 2 Material and methods

**Fig. 1:**
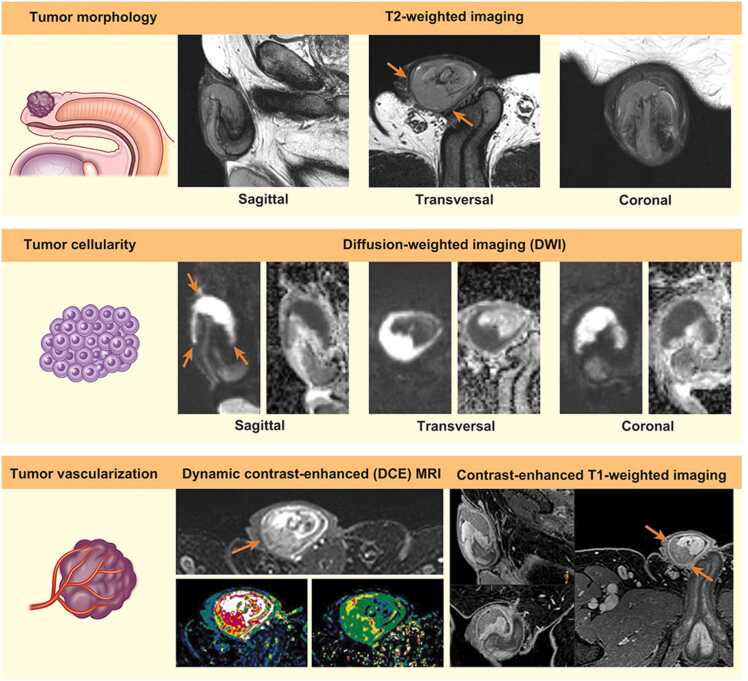
Principles of the multi-parametric magnetic resonance imaging protocol for preoperative evaluation of primary penile carcinoma. The interpretation is based on the integrated assessment of tumor morphology (upper row), cellularity (middle row), and vascularization (lower row).

**Fig. 2:**
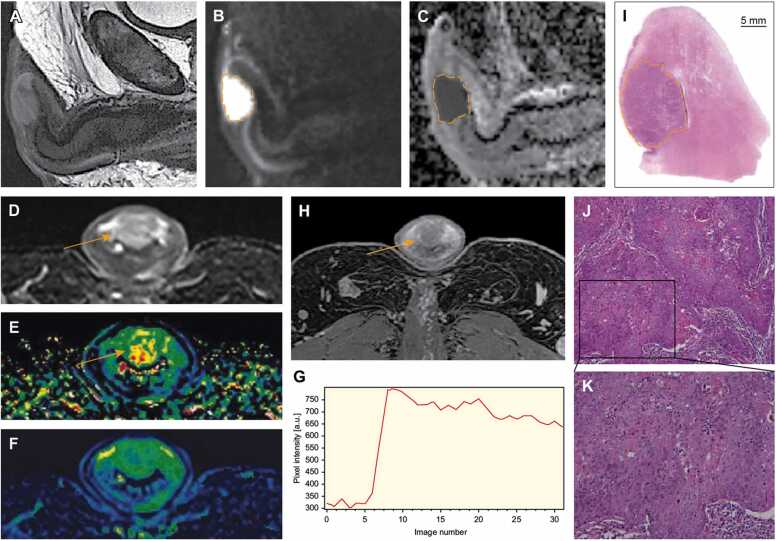
Preoperative multi-parametric magnetic resonance imaging (mpMRI) and histopathological findings in a patient with primary penile carcinoma. Sagittal T2-weighted sequence (A), diffusion-weighted imaging (B), and apparent diffusion coefficient (ADC) map (C) shows a large T2-tumor invading glans (dashed line). Tumor has homogenous, low diffusion (B, C) (ADC = 600 μmm^2^/s) and a rapid, initial permeability with wash-out on dynamic contrast-enhanced MRI perfusion (D–G; D, acquisition image; E, *K*^*trans*^; F, *k*_*ep*_; G, perfusion curve) (arrows), and delayed, contrast-enhanced T1-weighted Dixon sequence (H, arrow). Photomicrograph of whole-mount hematoxylin and eosin-stained section with outlined tumor invading glans/corpus spongiosum (I, dashed line). There was no involvement of tunica albuginea or corpus cavernosum. Photomicrographs of histologic specimen confirm infiltration of moderately to poorly differentiated keratinizing squamous cell carcinoma, usual type (J, magnification × 10; K, magnification × 20).

The study was approved by the Institutional Review Board (IRB) and the requirement for informed consent was waived for the retrospective reviewing of MRI and clinicopathological data of the included patients (IRB identifier: 22/09548). We retrospectively analyzed the imaging and clinicopathological data of 25 patients with primary penile SCC referred to our institution for surgery between May 2020 and June 2022. Preoperative mpMRI without artificial erection was performed in all patients. MRI was performed using a 3-T magnet (Vida Fit, Siemens) with a 32-channel phased-array coil. The imaging protocol consisted of high-resolution morphological and functional sequences covering the penis and lower pelvis (Table 1, Fig. 1, Fig. 2). The complete MRI protocol is described in Supplementary Table 1. All pretreatment MRI scans were retrospectively reviewed by a single radiologist (MDS) with 13 years of experience in urogenital radiology. T and N staging, according to the 8th edition of the Union for International Cancer Control TNM classification for penile cancer [18] as well as the largest diameter and thickness/infiltration depth of the primary lesions were determined for all patients. Since many tumors were large, exophytic, and displaced the anatomical structures, the tumor thickness/infiltration depth was measured from the tumor surface to the deepest point of invasion [19]. The lesions were outlined on apparent diffusion coefficient maps to minimalize the measurement of peritumoral edema and inflammation [20].

**Table 1:** Short overview of the multi-parametric magnetic resonance imaging protocol for preoperative assessment of primary penile carcinoma.

• The examination is performed without artificial erection. • Small FOV, high-resolution T2-weighted sequence in three anatomical planes (transversal, coronal and sagittal) over primary tumor. Sagittal T2-weighted sequence should cover the whole urethra. • Small FOV, high-resolution DWI in three anatomical planes (transversal, coronal and sagittal) over primary tumor. Sagittal DWI should cover the whole urethra. • Transversal large FOV DWI covering inguinal and pelvic lymph nodes. • Transversal dynamic contrast enhanced DCE-MRI perfusion over penis and urethra. • Transversal high-resolution 3D GE T1-weighted Dixon sequence after gadolinium over penis, urethra, inguinal and pelvic lymph nodes.

DWI = diffusion-weighted imaging. DCE-MRI = dynamic contrast-enhanced magnetic resonance imaging. FOV = field of view. GE = gradient echo.

Surgical approaches to penile and lymph node surgery were discussed within the institutional penile cancer team and performed according to the current guidelines [9]. Surgical approaches to penile surgery included radical circumcision, partial glans resection, glansectomy, partial penectomy, and total penectomy. The management of lymph nodes included surveillance, sentinel node biopsy (SNB), inguinal lymph node dissection, and pelvic lymph node dissection.

Surgical specimens were fixed in 10 % buffered formalin for at least 48 h, macroscopically examined, grossed, and embedded in the paraffin blocks. Sections were cut to a thickness of 4 µm and routinely stained with hematoxylin and eosin. The specimens were analyzed according to standardized protocols based on the American Joint Committee on Cancer format [21]. The largest tumor diameter and tumor infiltration depth/thickness were measured and the tumors were classified into subtypes and staged according to the TNM classification [18], [21].

The data were analyzed using IBM SPSS Statistics (version 28; IBM Corp., Armonk, NY, USA). Descriptive statistics including frequency distributions and percentages were used to describe the study population. Nonparametric Spearman rank-order correlation was applied to the continuous data. A correlation coefficient (ρ) of 0.90–1.00 implied a very strong correlation; 0.70–0.89, strong correlation; 0.40–0.69, moderate correlation; 0.10–0.39, weak correlation; and < 0.10, negligible correlation [22]. The relationship between categorical variables was analyzed using the Fisher-Freeman-Halton exact test. Kappa (κ) was estimated to evaluate the concordance between the imaging and histopathological features. A κ-values of 0.81–1.00 implied very good agreement; 0.61–0.80, good agreement; 0.41–0.60, moderate agreement; 0.21–0.40, fair agreement; and < 0.20, poor agreement [23]. Statistical tests were two-sided and a *p*-value < 0.05 was considered statistically significant.

## 3 Results

The age of the included patients ranged from 48 to 80 years, with a mean age of 68 years. Partial and total penectomies were the most frequent surgical modalities, accounting for 11 (44 %) and five (20 %) of the total cases, respectively. Other surgical approaches included glansectomy in four (16 %), partial glans resection in three (12 %), and radical circumcision in two (8 %) patients. Nineteen (76 %) patients were diagnosed with usual SCC, two (8 %) with sarcomatoid SCC, two (8 %) with basaloid SCC, and one (4 %) with papillary or verrucous SCC. A warty-basaloid SCC was found in one patient (4 %). The tumor was located on the glans in 15 (60 %) patients; on preputium in two (8 %); on preputium and glans in five (20 %), on glans and sulcus coronarius in two (8 %); and on preputium, glans, and sulcus coronarius in one (4 %) patient. According to the TNM classification, three (12 %) cases were classified as pathological (p) stage T1 (pT1), 18 (72 %) as pT2, and four (16 %) as pT3. Fourteen (56 %) patients had no lymph node metastases on histopathological evaluation or had a negative SNB (pN0), three (12 %) were classified as pN1, and three (12 %) as pN3. None of the patients were classified as pN2. Histopathological data were not available for five (20 %) patients (pNx). One (4 %) patient presented with a separate tumor satellite in the proximal corpus cavernosum.

A significant association was observed between MRI and histopathology for the involvement of anatomical structures (Table 2). A very good agreement was observed between MRI and histopathology for the involvement of corpus spongiosum (κ = 0.834, *p* = 0.002). Good agreement was observed between MRI and histopathology for the involvement of penile urethra and tunica albuginea/corpus cavernosum (urethra: κ = 0.746, *p* < 0.001; corpus cavernosum: κ = 0.702, *p* = 0.007).

**Table 2:** Comparison of magnetic resonance imaging and histopathological findings.

Histopathology	MpMRI						*p*-value	Kappa (κ)
Involvement of corpus spongiosum (n = 25)	Yes			No				
Yes	21 (84)			1 (4)			0.002	0.834
No	0 (0)			3 (12)				
Involvement of urethra (n = 24)^a^	Yes			No				
Yes	12 (50)			1 (4)			<0.001	0.746
No	2 (8)			9 (38)				
Involvement of corpus cavernosum (n = 25)	Yes			No				
Yes	3 (12)			1 (4)			0.007	0.702
No	1 (4)			20 (80)				
T staging (n = 25)	T1		T2		T3			
T1	3 (12)		0 (0)		0 (0)		<0.001	0.742
T2	1 (4)		16 (64)		1 (4)			
T3	0 (0)		1 (4)		3 (12)			
N staging (n = 20)^b^	N0	N1		N2		N3		
N0	12 (60)	1 (5)		1 (5)		0 (0)	0.002	0.606
N1	1 (5)	2 (10)		0 (0)		0 (0)		
N2	0 (0)	0 (0)		0 (0)		0 (0)		
N3	0 (0)	0 (0)		1 (5)		2 (10)		

Categorical variables are described by number (%) of patients. Percentages may not add up to 100 because of rounding. MpMRI = multi-parametric magnetic resonance imaging.

a Histopathological data not available for one patient.

b Histopathological data not available for five patients.

Good agreement was observed between MRI and histopathology for overall T staging (κ = 0.742, *p* < 0.001) (Table 2). Twenty-two (88 %) patients were correctly staged on MRI, one (4 %) patient was overstaged (mrT3 vs. pT2), and two (8 %) patients were understaged (mrT1 vs. pT2, mrT2 vs. pT3) when compared with histopathological staging.

Good agreement was observed between MRI and histopathology for N staging (κ = 0.606, *p* = 0.002) (Table 2). Histopathological data were not available for five (20 %) patients (pNx). Sixteen (80 %) of 20 patients with available histopathological data were correctly staged, two (10 %) patients were overstaged (mrN1 vs. pN0, mrN2 vs. pN0), and two (10 %) patients were understaged on MRI (mrN0 vs. pN1, mrN2 vs. pN3). Nodal metastases were missed on MRI in one (5 %) patient.

**Fig. 3:**
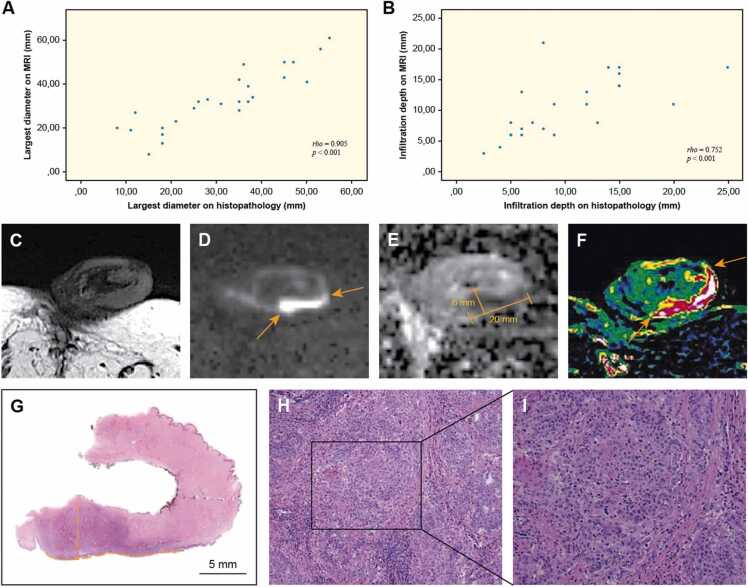
Scatterplot demonstrates comparison of largest tumor diameter (A) and infiltration depth (B) as determined by multi-parametric magnetic resonance imaging (mpMRI) and histopathology. Correlation coefficient (ρ) and *p*-value from Spearman rank-order correlation are shown. An example on correlation between mpMRI findings (C-F) and histopathology is also shown (G–I). Tumor is difficult to outline on T2-weighted sequence (C). However, diffusion-weighted sequence (D) and apparent diffusion coefficient map (E) clearly illustrate tumor extension (arrows). Tumor extension is larger on *K*^*trans*^ perfusion map (F), probably due to peritumoral edema and inflammation. Photomicrograph of whole-mount hematoxylin and eosin-stained section with 6 × 18 mm T2-tumor invading glans (G, dashed lines). Photomicrographs of histologic specimen confirm infiltration of poorly differentiated keratinizing squamous cell carcinoma, usual type (H, magnification × 10; I, magnification × 20).

A strong and significant correlation was observed between MRI and histopathology for the largest diameter and thickness/infiltration depth of the primary lesions (Table 3, Fig. 3) (largest diameter: ρ = 0.905, *p* < 0.001; tumor thickness/infiltration depth: ρ = 0.752, *p* < 0.001).

**Table 3:** Comparison of tumor size and infiltration depth on magnetic resonance imaging and histopathology.

Variable	MpMRI	Histopathology	*p-*value	Spearman’s coefficient (ρ)
Tumor size (mm)				
Largest diameter Thickness/infiltration depth^a^	33.2 (8–61) 10.7 (3–21)	31.2 (8–55) 10.3 (2.5–25)	< 0.001 < 0.001	0.905 0.752

Continuous variables are described by mean (range). MpMRI = multi-parametric magnetic resonance imaging.

a Histopathological data not available for two patients.

## 4 Discussion

The present study reported the initial experience of non-erectile mpMRI for the preoperative assessment of primary penile carcinoma. We found good agreement between MRI and histopathology for T and N staging, and a strong and significant correlation between MRI and histopathology for the largest tumor diameter and infiltration depth.

Few reports have assessed the diagnostic potential of MRI in preoperative evaluation of penile carcinomas; however, inconsistencies in MRI protocols can influence the outcomes and make comparisons difficult [13]. In our study, good agreement was observed between MRI and histopathology for T staging and was similar to or higher than that reported previously [2], [10], [11], [24], [25], [26], including studies wherein MRI was performed after pharmacological erection [2], [10], [11], [26]. We observed a weaker, but still good agreement between MRI and histopathology for N staging. This was anticipated because MRI, even with functional sequences, has limited sensitivity in detecting small lymph node metastases and small amounts of tumor within a lymph node [27]. A few prior studies have investigated the value of MRI in assessing the N status in penile cancer and the outcomes are mostly comparable to our results [25], [28].

Most of the studies assessing MRI for the local staging of penile cancers used standard, morphological sequences alone, and only a few studies included DCE-MRI perfusion or DWI in the preoperative protocols [2], [24], [26], but none of them combined both DCE-MRI and DWI to a high-resolution mpMRI. Morphological sequences tend to overestimate the tumor extent because peritumoral edema and hyperemia are difficult to differentiate from the tumor tissue on these sequences [20]. Functional sequences seem to depict tumor tissue more selectively, and despite lower spatial resolution, delineate the tumor extent more precisely. DWI visualizes the high cellularity of the tumor as opposed to the surrounding tissue, and early phases of DCE-MRI perfusion visualize the enhancement in the intratumoral vessels before the contrast medium reaches the peritumoral vessels [16], [29].

Our results showed a strong and significant correlation between MRI and histopathology for the largest tumor diameter and infiltration depth (Fig. 3). A study by Lont et al. investigated the role of imaging in determining the extent and infiltration depth of primary penile carcinomas [30]. They concluded that physical examination is the most reliable method for determining tumor size, while MRI was assessed to be less precise. However, this study was published 20 years ago, and comparing its results with those of the current study is, to a large extent, not possible due to principal differences in MRI protocols and anticipated low spatial resolution of images. Our findings are encouraging for implementing mpMRI as a precise diagnostic method for surgical planning. The ability to precisely estimate tumor thickness and depth of invasion is important, since infiltration depth is one of the prognostic factors for the development of metastases in non-verruciform tumors invading the corpus spongiosum [31]. Tumors invading superficially (< 5 mm) in the corpus spongiosum rarely metastasize, while tumors showing deep invasion in the corpus spongiosum (> 5 mm) are associated with a higher rate of metastasis, similar to those invading the corpus cavernosum [19], [31]. Furthermore, a vast majority of early penile carcinomas are amenable to organ-sparing techniques [10]. Accurate outlining of the tumor extent and appropriate preoperative staging can thus improve patient selection and decrease the recurrence rate after OSS.

The main limitations of this study are its retrospective design and limited sample size. The main strengths of the study are its high technical image quality and thorough histopathologic correlation. Despite the limited number of patients in our pilot analysis, the agreement between imaging and histopathological features was remarkably consistent. To verify these findings, a prospective study on a large dataset of patients with penile cancer, correlating mpMRI and 2-fluoro-2-deoxy-D-glucose positron emission tomography/computed tomography (FDG PET-CT) with clinicopathological features has been initiated (ClinicalTrials.gov identifier: NCT05447273).

## 5 Conclusions

Good concordance was observed between the MRI and histopathological findings despite the small sample size. Our initial findings indicate that mpMRI without artificial erection can be implemented as a precise diagnostic tool for preoperative assessment of primary penile cancer. However, the results should be verified in a larger and, preferably, prospectively collected dataset.

## CRediT authorship contribution statement

**MDS:** Conceptualization, Methodology, Formal analysis, Investigation, Writing – original draft, Visualization, Project administration, Writing – review & editing. **AH:** Methodology, Formal analysis, Investigation, Writing – original draft, Writing – review & editing. **SS:** Formal analysis, Investigation, Writing – original draft, Writing – review & editing. **BB:** Investigation, Supervision, Writing – review & editing. **FO:** Investigation, Writing – review & editing. **KB:** Writing – review & editing. **UA:** Investigation, Writing – review & editing. **KHH:** Investigation, Writing – review & editing, Supervision.

## Ethical statement

The Institutional Review Board approved this retrospective study. The need for written informed consent was waived.

## Funding

This research did not receive any specific grant from funding agencies in the public, commercial, or not-for-profit sectors.

## Declaration of Competing Interest

The authors declare that they have no known competing financial interests or personal relationships that could have appeared to influence the work reported in this paper.
