# Supplementary material for: Multi-parametric MRI without artificial erection for preoperative assessment of primary penile carcinoma: A pilot study on the correlation between imaging and histopathological findings
Source: Eur J Radiol Open. 2023 Jan 28;10:100478. doi: 10.1016/j.ejro.2023.100478 (PMC9922909; doi:10.1016/j.ejro.2023.100478)
Supplement: Supplementary file 1 — Supplementary material [file mmc1.docx]

**Supplementary Table 1.** Multi-parametric magnetic resonance imaging protocol for the preoperative assessment of primary penile carcinoma (3-T Vida Fit, Siemens)

| **MpMRI parameters** | **T2W** | **DWI RESOLVE** | **DWI** | **DCE-MRI** | **T1W after Gd** |
| --- | --- | --- | --- | --- | --- |
| Pulse sequence | 2D SE | Small FOV SE-EPI | STIR-EPI | 3D spoiled GE Dixon | 3D spoiled GE Dixon |
| Coverage area | Primary tumor | Primary tumor | Primary tumor, inguinal and pelvic lymph nodes | Primary tumor | Primary tumor, inguinal and pelvic lymph nodes |
| Plane | Sag, Cor, Tra | Sag, Cor, Tra | Tra | Tra | Tra |
| Slice thickness (mm)  Acquired  Interpolated | 2.0 | 2.3 | 5.0 | 2.2  1.1 | 1.2  0.6 |
| FOV (mm) | 230 | 150 | 370 | 169 | 306 |
| TE (ms) | 99 | 56 | 64 | 1.35 and 2.58 | 2.46 and 3.69 |
| TR (ms)  TI (ms) | 2430  - | 2910  - | 7190  240 | 4.4  - | 5.8  - |
| Flip angle | 128° | 180° | - | 13° | 12° |
| Gap (mm) | 0.0 | 0.8 | 1.0 | 0.44 | 0.24 |
| NEX | 2 | b0 = 2  b800 = 4 | b0 = 3  b800 = 3 | 1 | 2 |
| In-plane resolution (mm x mm)  Acquired  Interpolated | 0.72 x 0.72  0.36 x 0.36 | 1.47 x 1.47 | 2.5 x 2.5  1.25 x 1.25 | 1.32 x 1.32  0.66 x 0.66 | 0.82 x 0.74  0.37 x 0.37 |
| ETL | 17 | 69 | 120 | - | - |
| Bandwidth (Hz/Px) | 313 | 980 | 2112 | 810 | 670 |
| Matrix  Acquired  Interpolated | 320 x 320  640 x 320 | 102 x 102 | 148 x 148  296 x 296 | 128 x 128  256 x 256 | 416 x 374  832 x 748 |
| R-factor | 2 | 2 | 2 | 4 | 4 |
| b-values | - | Acquired 0 and 800, calculated 1400 | Acquired 0 and 800 | - | - |
| Time resolution (sec) | - | - | - | 9.38 | - |
| Acquisition time (min) | 2:52 | 3:40 | 2:17 | 5:12 | 6:05 |

MpMRI = multi-parametric magnetic resonance imaging. DWI = diffusion-weighted imaging. DCE-MRI = dynamic contrast-enhanced magnetic resonance imaging. Gd = gadolinium. SE = spin echo. FOV = field of view. EPI = echo-planar imaging. STIR = short tau inversion recovery. GE = gradient echo. Sag = sagittal. Cor = coronal. Tra = transversal. TE = echo time. TR = resolution time. TI = inversion time. NEX = number of excitations. ETL = echo train length.
